# Short-term effects of different toothpaste formulations on total salivary calcium: A randomized crossover study

**DOI:** 10.1371/journal.pone.0358715

**Published:** 2026-09-17

**Authors:** Lina Aminy, Mozhgan Bizhang, Claudia Klinger, Stefan Zimmer, Andreas Savelsbergh

**Affiliations:** 1 Chair of Operative and Preventive Dentistry, Witten/Herdecke University, Witten, Germany; 2 Division of Functional Genomics, Chair of Biochemistry and Molecular Medicine, ZBAF, Witten/Herdecke University, Witten, Germany; Mersin University: Mersin Universitesi, TÜRKIYE

## Abstract

**Objectives:**

Dental calculus formation can be inhibited by toothpastes containing pyrophosphate, which chelates calcium ions and prevents hydroxyapatite crystal formation. Although pyrophosphate may also influence salivary calcium availability, the short-term effects of commercially available toothpaste formulations on salivary calcium concentrations remain insufficiently characterized. This study investigated and compared the short-term effects of three fluoride toothpastes on salivary calcium concentration.

**Methods:**

A randomized, three-treatment, three-period crossover trial was conducted in 48 healthy volunteers. The participants used three commercially available sodium fluoride toothpastes in randomized order: a formulation containing methyl vinyl ether/maleic acid copolymer, PVM/MA copolymer (Sensodyne), a formulation containing pyrophosphate and zinc (Dentalux), and a formulation containing sodium bicarbonate (Parodontax). Treatment periods were separated by a 2-day washout. Unstimulated saliva was collected at baseline (T0), immediately after brushing (T1), 30 min (T2), and 60 min (T3). Salivary calcium concentrations were quantified using a colorimetric assay. Data were analysed using linear mixed-effects models with toothpaste formulation, measurement time, treatment sequence, and the toothpaste-by-time interaction as fixed effects and participant as a random effect.

**Results:**

Significant effects were observed for toothpaste formulation (F(2,103.35)=42.31, P < 0.001), measurement time (F(3,141)=39.78, P < 0.001), and the toothpaste-by-time interaction (F(6,141)=23.91, P < 0.001), whereas treatment sequence had no significant effect (P = 0.927). Compared with Dentalux® and Sensodyne®, Parodontax® resulted in significantly lower salivary calcium concentrations (both Bonferroni-adjusted P < 0.001), immediately after brushing (T1). The differences decreased after 30 min and were no longer significant after 60 min. No significant differences were observed between Dentalux® and Sensodyne® at any time point.

**Conclusions:**

Commercially available fluoride toothpastes produced different short-term effects on the salivary calcium concentration, with the greatest differences occurring immediately after brushing. Because the products differed in terms of multiple formulation ingredients, the findings reflect product-level effects rather than ingredient-specific effects. Further multicentre studies are warranted to confirm these findings and evaluate their clinical relevance.

## Introduction

Toothpaste provides multiple oral health benefits, including improved oral freshness, anticariogenic efficacy, inhibition of biofilm regrowth, tooth whitening, and enhanced patient compliance, largely due to the incorporation of chemotherapeutic agents [1]. Dental calculus is a mineralized biofilm composed of an inorganic mineral phase embedded within an organic matrix. Its mineral components consist primarily of brushite (dicalcium phosphate dihydrate), octacalcium phosphate (OCP), hydroxyapatite (HAP), and whitlockite. Calculus formation begins with the acquired pellicle, a protein-rich salivary layer that rapidly forms on the enamel surface and facilitates bacterial adhesion and subsequent biofilm development. As the biofilm matures, calcium and phosphate ions precipitate within the extracellular matrix, leading to progressive mineralization and calculus formation [2–4]. Saliva plays a central role in oral homeostasis and is essential for both calculus formation and enamel remineralization. Its ionic composition resembles that of systemic body fluids, while its buffering capacity helps maintain a stable oral pH. In addition, saliva contains calcium, phosphate, and other inorganic ions that promote the remineralization of early carious lesions and previously demineralized enamel, thereby constituting an intrinsic defence mechanism against mineral loss [5]. Because calculus formation depends on calcium phosphate precipitation, the availability of free calcium ions in saliva is a key determinant of mineral deposition and may influence remineralization processes.

Although mechanical plaque removal remains the cornerstone of oral hygiene, chemotherapeutic agents are widely incorporated into toothpastes to inhibit plaque mineralization and calculus formation. Chelating agents reduce calcium availability by forming soluble complexes, thereby interfering with crystal nucleation and growth. Soluble pyrophosphates are among the most extensively studied anti-calculus agents and have consistently been shown to inhibit mineral deposition, delay hydroxyapatite formation, and retard the phase conversion from brushite to hydroxyapatite [6–10]. Zinc salts further inhibit plaque mineralization without compromising the anticaries activity of fluoride and are therefore frequently combined with pyrophosphates [10–12] Another widely used anti-calculus agent is methyl vinyl ether/maleic acid copolymer (PVM/MA, Gantrez^®^), which reduces bacterial adhesion by modifying enamel surface properties and decreasing calcium availability while also exerting modest inhibitory effects on crystal growth [13–15]

Whether anticalculus formulations influence fluoride-mediated remineralization remains controversial. Several in vitro and experimental studies have reported no significant effects of pyrophosphate-containing toothpastes on fluoride uptake or enamel remineralization [15–18]. In contrast, long-term clinical trials have consistently demonstrated greater reductions in caries progression with conventional sodium fluoride toothpastes than with randomized stannous fluoride/pyrophosphate formulations [19–21], whereas randomized controlled trials directly comparing fluoride toothpastes with and without pyrophosphates remain scarce and have yielded inconsistent findings [12]. Consequently, the current evidence does not conclusively exclude the possibility that pyrophosphate-containing formulations may reduce anticaries efficacy. From a mechanistic perspective, pyrophosphates inhibit calcium phosphate nucleation and hydroxyapatite crystal growth, providing a biologically plausible explanation for the potential reduction in remineralization. Moreover, the reported inverse association between calculus accumulation and caries incidence offers indirect evidence for the hypothesis that strategies designed to inhibit calculus formation may unintentionally influence caries development [22]. Although research on toothpaste formulations has increasingly shifted towards other active ingredients, these unresolved questions warrant renewed investigation [23].

To date, little is known about the immediate effects of anticalculus toothpastes on salivary calcium availability. Since pyrophosphate acts primarily through calcium complexation, measuring salivary calcium concentrations may provide valuable insight into the biological availability of calcium following toothpaste use and its potential implications for remineralization.

Therefore, the present randomized three-period crossover study was designed to evaluate the effects of a single application of three toothpastes—two containing anticalculus agents (pyrophosphate/zinc and PVM/MA) and one without anticalculus agents—on salivary calcium concentrations. The primary objective was to determine whether changes in salivary calcium concentration over time differed between toothpaste formulations. The secondary objectives were to compare salivary calcium concentrations across toothpaste formulations, measurement times, and treatment sequences.

## Materials and methods

### Ethics

Ethical approval was obtained from the Ethics Committee of Witten/Herdecke University (S-324/2023). The study was conducted in accordance with the principles of the Declaration of Helsinki. Before participation, all participants received detailed information about the study and provided written informed consent before enrolment. No minors were included in the study.

Trial registration: This study was originally designed as an exploratory physiological crossover study investigating salivary biomarkers as the primary outcome, without evaluating clinical efficacy endpoints. Hence prospective trial registration was not performed before participant enrolment. To ensure transparency, the study was retrospectively registered in the German Clinical Trials Register (DRKS) under the identifier DRKS00039385.

### Sample size

Power analysis was conducted using G*Power (version 3.1) [24]. An a priori sample size calculation for a repeated-measures design indicated that a total sample size of 48 participants would be required to detect a medium effect size (f = 0.25) with 80% statistical power at α = 0.05, assuming four repeated measurements and a correlation of 0.50 among repeated observations (achieved power = 0.804). Accordingly, 48 participants were enrolled in this randomized crossover trial.

### Study design

The investigation was designed as a randomized, three-treatment, three-period crossover clinical trial. Each participant received all three toothpaste formulations according to one of three predefined treatment sequences (ABC, BCA, or CAB) (Table 1). Each treatment period was separated by a 48-hour washout period. Before the first treatment period, all participants completed a seven-day run-in phase in which they used a fluoride- and additive-free toothpaste (Ajona®, Dr. Liebe GmbH & Co. KG, Germany) to minimize potential carry-over from previously used oral hygiene products. The primary endpoint was the toothpaste-by-time interaction in the salivary calcium concentration after a single toothbrushing procedure. Secondary endpoints included the main effects of toothpaste formulation, measurement time, treatment sequence, and Bonferroni-adjusted pairwise comparisons between toothpaste formulations.

**Table 1:** Randomized crossover study design showing the treatment sequences. Participants were allocated to one of three sequences (ABC, BCA, or CAB) and received the three test toothpastes (Dentalux, Parodontax, and Sensodyne) in different orders. Each treatment period was separated by a two-day washout period.

Sequence	Period 1	Washout	Period 2	Washout	Period 3
ABC	Dentalux	2 d	Parodontax	2 d	Sensodyne
BCA	Parodontax	2 d	Sensodyne	2 d	Dentalux
CAB	Sensodyne	2 d	Dentalux	2 d	Parodontax

### Participants

Participants were recruited between April 21, 2024, and December 6, 2024, from the students of Witten/Herdecke University. Eligible individuals were in good general and oral health and had at least 20 natural permanent teeth, a stimulated whole salivary flow rate ≥ 0.8 mL/min, and an unstimulated whole salivary flow rate ≥ 0.5 mL/min. Stimulated and unstimulated salivary flow rates were determined at the screening visit to verify eligibility. Salivary flow rate and salivary pH were not measured during the experimental visits because the primary objective of the study was to compare short-term changes in salivary calcium concentration under standardized experimental conditions. Nevertheless, both parameters may influence calcium concentrations and are therefore acknowledged as study limitations.

The exclusion criteria included pregnancy or breastfeeding; chronic systemic disease or conditions associated with xerostomia; diabetes; known intolerance or hypersensitivity to study materials; antibiotic use at the time of screening or within the preceding two weeks; and the use of multivitamins, calcium, or fluoride supplements within seven days prior to the study phase. Dental-related exclusion criteria comprised untreated caries; severe periodontal disease, defined as a Periodontal Screening Index (PSI) score ≥3 in at least one sextant; oral lesions; ulcers; inflammatory conditions; and dental treatment during the study period. All individuals who fulfilled the predefined eligibility criteria and provided written informed consent were enrolled until the required sample size (n = 48) was reached.

### Randomization

Participants were randomly assigned to one of three treatment sequences (ABC, BCA, or CAB) using a computer-generated randomization list prepared before enrolment by a study member who was not involved in participant recruitment or laboratory analyses.

**Fig 1:**
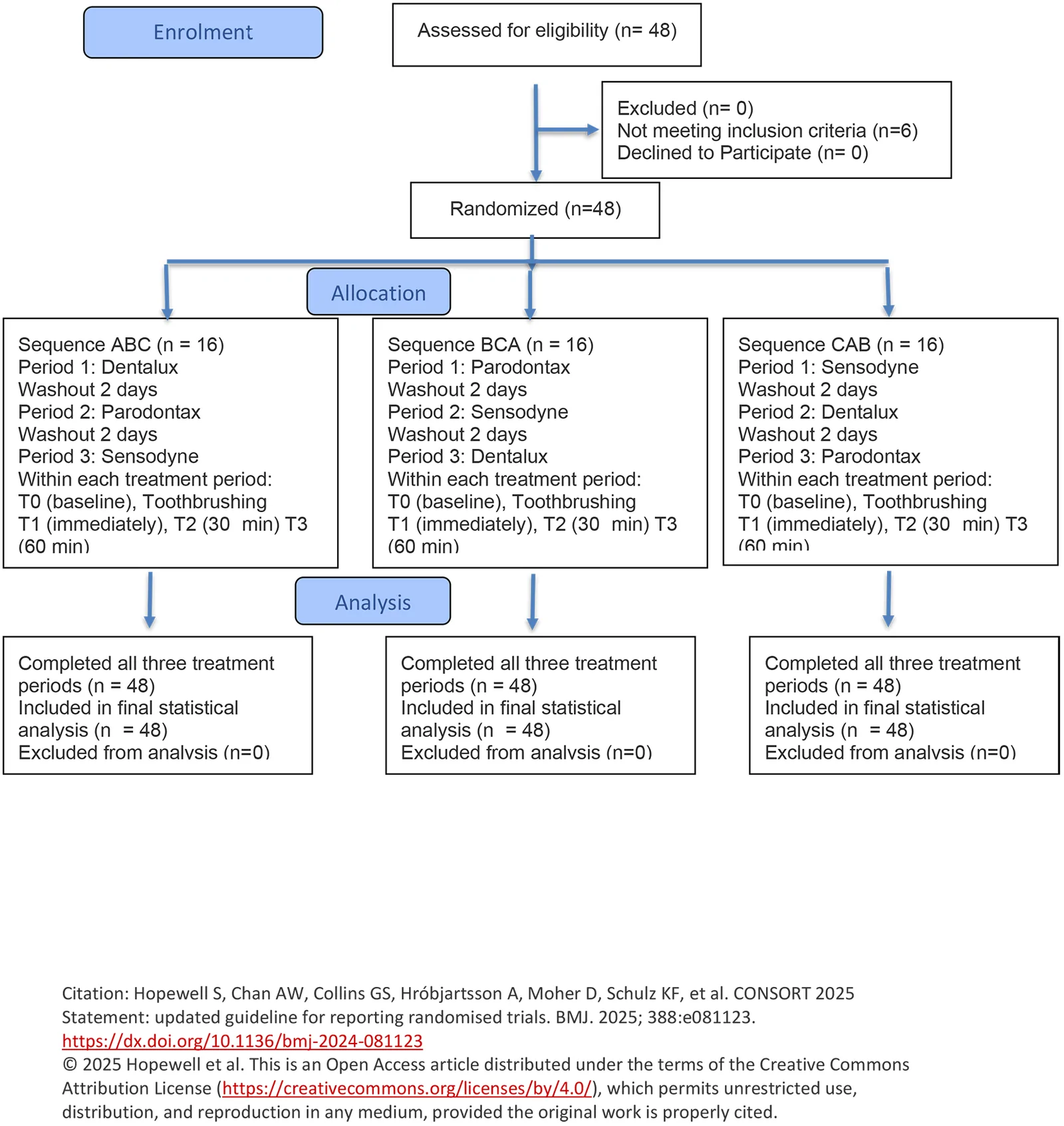
CONSORT flow diagram showing participant recruitment, randomization to the three crossover treatment sequences (ABC, BCA, and CAB), 48-hour washout periods between treatment periods, completion of follow-up, and inclusion in the final statistical analysis.

Allocation concealment was ensured by sequentially numbered, opaque, sealed envelopes that were opened only after participant enrolment. The investigator responsible for screening and recruitment had no access to the randomization schedule before allocation (Fig 1).

### Intervention

The study was conducted by L.A. at the university laboratory. Baseline saliva samples (T0) were obtained prior to tooth brushing. Participants then brushed their teeth using one of the three assigned toothpaste formulations (Table 2).

**Table 2:** Toothpastes used in the study and their ingredients.

Products	Ingredients
A: Lidl – Dentalux Triple Protection Fresh Gel	Sorbitol, Aqua, Hydrated Silica, Sodium C14-16 Olefin Sulfonate, Aroma, Cellulose Gum, Glycerin, Propylene Glycol, Tetrapotassium Pyrophosphate, Sodium Fluoride, Sodium Saccharin, Zinc Chloride, CI 42090. Contains sodium fluoride (1.450 ppm fluoride)
B: Parodontax	Sodium Bicarbonate, Aqua, Glycerin, Cocamidopropyl Betaine, Aroma, Xanthan Gum, Sodium Fluoride, Sodium Saccharin, Steviol Glycosides, Limonene, CI 77491. Contains sodium fluoride (1.400 ppm F-).
C: Sensodyne ProSchmelz Repair	Aqua, Sorbitol, Hydrated Silica, Glycerin, Potassium Nitrate, PEG-6, Sodium Lactate, Cocamidopropyl Betaine, Aroma, Titanium Dioxide, Xanthan Gum, Sodium Saccharin, Sodium Fluoride, PVM/MA Copolymer, Sodium Hydroxide, Limonene. Contains sodium fluoride (1.450 ppm fluoride).

All participants used the same manual toothbrush and brushed according to the Stillman technique for two minutes under the supervision of an investigator. Participants were instructed to refrain from eating or drinking for two hours before each study visit, except for still water. Immediately after tooth brushing, saliva samples were collected (T1), followed by additional collections at 30 minutes (T2) and 60 minutes (T3). Following completion of each treatment period, participants entered a two-day washout period before receiving the next toothpaste according to the randomized crossover sequence (Table 3).

**Table 3:** Study protocol and experimental timeline.

Toothpaste Dentalux (D) (NaF + Pyrophosphate + Zinc) n = 16	Toothpaste Parodontax (P) (NaF, sodium bicarbonate) n = 16	Toothpaste Sensodyne (S) (NaF + PVM/MA) n = 16
Wash-out-Phase Seven days of using a fluoride- and additive-free toothpaste (Ajona)		
• Saliva collection (T0–T3) T0: before toothbrushing T1: immediately after toothbrushing T2: 30 minutes after T3: 60 minutes after	• Saliva collection (T0–T3) T0: before toothbrushing T1: immediately after toothbrushing T2: 30 minutes after T3: 60 minutes after	• Saliva collection (T0–T3) T0: before toothbrushing T1: immediately after toothbrushing T2: 30 minutes after T3: 60 minutes after
Two-day washout phase (crossover)		

### Saliva collection

Saliva samples were collected using Salivette® collection devices (Sarstedt AG, Nümbrecht, Germany). Each collection period lasted five minutes. The samples were centrifuged immediately after collection and stored at −80°C for laboratory analysis. All the samples were pseudonymized using unique study identification numbers. The linkage file connecting participant identities with study IDs was stored separately in a password-protected electronic file accessible only to the principal investigator.

### Calcium assay

Calcium concentrations were determined using a colorimetric calcium assay kit (Abcam, Catalogue No. ab102505, Cambridge, UK) according to the manufacturer’s instructions. The samples were thawed at 4°C, centrifuged at 1,000 × g, and 50 µL aliquots were transferred to 96-well microplates together with duplicate standards and blank controls. Following the addition of calcium assay buffer and a chromogenic reagent, the plates were incubated for 10 minutes in the dark at room temperature. Absorbance was measured at 575 nm using a SpectraMax® ABS Plus microplate reader (Molecular Devices, San Jose, CA, USA). Duplicate measurements were averaged, and the blank absorbance was subtracted before interpolation from the standard curve.

Calcium concentration = (Sa/Sv) × D

where Sa represents the calcium amount (µg), Sv represents the analysed sample volume (µL), and D represents the dilution factor. Since saliva has a density close to 1 g/mL, calcium concentrations are reported in ppm, which is equivalent to mg/L for aqueous biological fluids.

### Data analysis

Statistical analyses were performed using IBM SPSS Statistics for Windows, Version 29 (IBM Corp., Armonk, NY, USA). Model assumptions were evaluated by visual inspection of standardized residual histograms, normal probability (Q–Q) plots, and residual-versus-fitted plots. Residual diagnostics revealed no substantial deviations from normality or homoscedasticity. Because the study employed a randomized three-treatment, three-period crossover design with repeated measurements, salivary calcium concentrations were analysed using linear mixed-effects models (LMMs) estimated by restricted maximum likelihood (REML). This approach appropriately accounts for repeated observations within participants and the hierarchical structure of the data. Salivary calcium concentration (Ca, ppm) was specified as the dependent variable. Toothpaste formulation, measurement time, treatment sequence, and the interaction between toothpaste formulation and measurement time were included as fixed effects. Participant identification number (ID) was included as a random intercept to account for repeated observations within individuals. Repeated measurements were modelled using an unstructured covariance matrix. A treatment sequence was included to evaluate potential sequence effects. Because the treatment sequence and study period were partially confounded by the three-sequence crossover design, period effects were evaluated separately in sensitivity analyses rather than simultaneously with sequence to avoid model collinearity. A two-day washout period was implemented between treatment periods to minimize potential carry-over effects. The global effects of toothpaste formulation, measurement time, treatment sequence, and the toothpaste×time interaction were assessed using Type III tests of fixed effects. Estimated marginal means (EMMs) together with their corresponding 95% confidence intervals (95% CIs) were calculated for each toothpaste formulation and measurement time. Pairwise comparisons between toothpaste formulations at each time point were adjusted for multiple testing using the Bonferroni procedure. Since each participant received all three toothpaste formulations, all treatment comparisons were based on within-subject estimates generated by the mixed-effects model. Therefore, independent-samples tests were not performed in the revised analysis. In addition to statistical significance, estimated effect sizes, adjusted mean differences, and their corresponding 95% confidence intervals were considered when the magnitude and precision of the observed treatment effects were interpreted. All statistical tests were two-sided, and a P value ≤ 0.05 was considered statistically significant.

## Results

A total of 48 participants were included in the final statistical analysis. The mean age of the study population was 22.55 ± 2.42 years. The study cohort consisted of 26 women (54.2%) and 22 men (45.8%). No participants were excluded after allocation, and all individuals completed the study protocol, with measurements taken at all time points. All randomized participants completed all three treatment periods and all scheduled saliva samples were collected (T0–T3). Consequently, no outcome data were missing, and all participants were included in the primary analysis.

Model assumptions were assessed by visual inspection of residual histograms, normal Q–Q plots, and residual vs fitted plots. No substantial deviations from normality or homoscedasticity were observed.

### Linear mixed-effects model

The mean salivary calcium concentrations (ppm) with corresponding 95% confidence intervals (CIs) for each toothpaste formulation at each measurement time are presented in Table 4.

**Table 4:** Mean salivary calcium concentrations (ppm) according to toothpaste formulation and measurement time (T0–T3).

Toothpaste	T0 Mean 95% CI)	T1 Mean (95% CI)	T2 Mean (95% CI)	T3 Mean (95% CI)
Dentalux (NaF + Pyrophosphate+ zinc)	38.9 (37.6–40.2)	36.6 (34.9–38.3)	39.2 (37.5–40.8)	39.4 (37.8–41.0)
Parodontax (NaF + Sodium bicarbonate)	37.5 (35.0–40.0)	18.8 (15.7–21.9)	35.8 (33.3–38.3)	36.9 (34.7–39.2)
Sensodyne (NaF + PVM/MA)	38.1 (36.4–39.7)	37.7 (35.8–39.6)	39.7 (38.5–40.9)	37.4 (35.6–39.2)

The values are presented as observed means with corresponding 95% confidence intervals.

Treatment sequence was included in the model to account for potential sequence effects. Because of the characteristics of the three-sequence Latin-square crossover design, the period and treatment sequence were collinear and could not be entered simultaneously into the model. Therefore, the treatment sequence was retained in the final model, whereas the potential influence of period and carry-over effects was evaluated through sensitivity analyses. No evidence of clinically meaningful carry-over effects was identified. Parameter estimates of the linear mixed-effects model are presented in Table 5. The reference categories were Sensodyne (toothpaste formulation), T3 (60 min after brushing), and sequence CAB.

**Table 5:** Fixed-effect parameter estimates from the linear mixed-effects model evaluating the effects of toothpaste formulation, measurement time, treatment sequence, and their interaction on salivary calcium concentration (ppm).

Fixed effect	Estimate (β)	SE	df	t	P value	95% CI
Intercept	37.48	1.34	77.9	28.00	<0.001	34.81 to 40.14
Dentalux vs. Sensodyne	1.97	1.11	110.2	1.77	0.079	−0.24 to 4.17
Parodontax vs. Sensodyne	−0.53	1.11	110.2	−0.47	0.637	−2.73 to 1.68
Time 0 vs. Time 3	0.61	1.08	141	0.57	0.573	−1.53 to 2.75
Time 1 vs. Time 3	0.26	1.29	141	0.20	0.839	−2.28 to 2.80
Time 2 vs. Time 3	2.30	0.84	141	2.73	0.007	0.64 to 3.97
Dentalux × Time 2	−2.56	1.19	141	−2.15	0.034	−4.92 to −0.20
Parodontax × Time 1	−18.36	1.82	141	−10.10	<0.001	−21.96 to −14.77
Parodontax × Time 2	−3.39	1.19	141	−2.85	0.005	−5.75 to −1.04
Sequence ABC vs. CAB	−0.36	1.58	41.8	−0.23	0.821	−3.55 to 2.83
Sequence BCA vs. CAB	0.25	1.58	41.8	0.16	0.875	−2.94 to 3.44

Abbreviations: β, fixed-effect regression coefficient; SE, standard error; df, denominator degrees of freedom; CI, confidence interval. Estimates were obtained from a linear mixed-effects model fitted by restricted maximum likelihood (REML). The reference categories were Sensodyne (toothpaste formulation), T3 (60 min after brushing), and sequence CAB. P values < 0.05 were considered statistically significant.

Type III tests of fixed effects demonstrated significant main effects of toothpaste formulation (F(2,103.35) = 42.31, P < 0.001) and measurement time (F(3,141.00) = 39.78, P < 0.001). A significant toothpaste-by-time interaction was also observed (F(6,141.00) = 23.91, P < 0.001), indicating that temporal changes in salivary calcium concentration differed among the toothpaste formulations. No significant treatment sequence effect was detected (F(2,41.77) = 0.08, P = 0.927), indicating that the order of toothpaste administration did not influence salivary calcium concentrations (Table 6).

**Table 6:** Type III tests of fixed effects from the linear mixed-effects model evaluating the effects of toothpaste formulation, measurement time, treatment sequence, and the toothpaste-by-time interaction on salivary calcium concentration (ppm).

Fixed effect	Numerator df	Denominator df	F	P value
Toothpaste	2	103.35	**42.31**	**<0.001**
Time	3	141.00	**39.78**	**<0.001**
Toothpaste × Time	6	141.00	**23.91**	**<0.001**
Sequence	2	41.77	0.08	0.927

Abbreviations: df, degrees of freedom; F, F statistic. The results are based on Type III tests of fixed effects from the linear mixed-effects model estimated by restricted maximum likelihood (REML). P values < 0.05 were considered statistically significant.

### Estimated marginal means

The estimated marginal means (EMMs) of salivary calcium concentration with corresponding 95% confidence intervals (CIs) derived from the linear mixed-effects model are presented in Table 7. After adjustment for repeated measurements and treatment sequence, Dentalux exhibited the highest overall adjusted mean salivary calcium concentration (38.51 ppm; 95% CI, 36.92–40.10), followed by Sensodyne (38.23 ppm; 95% CI, 36.64–39.82), whereas Parodontax showed the lowest adjusted mean concentration (32.26 ppm; 95% CI, 30.67–33.85).

**Table 7:** Estimated marginal means (EMMs) of salivary calcium concentration (ppm) with 95% confidence intervals according to toothpaste formulation and measurement time (T0–T3) derived from the linear mixed-effects model.

Toothpaste	Time	EMM (ppm)	SE	95% CI
Dentalux	T0	38.91	0.90	37.13–40.68
	T1	36.57	1.29	34.03–39.12
	T2	39.15	0.99	37.19–41.11
	T3	39.41	0.98	37.47–41.35
Parodontax	T0	37.51	0.90	35.74–39.29
	T1	**18.81**	1.29	16.27–21.36
	T2	35.82	0.99	33.86–37.78
	T3	36.91	0.98	34.97–38.85
Sensodyne	T0	38.05	0.90	36.28–39.83
	T1	37.70	1.29	35.16–40.25
	T2	39.74	0.99	37.78–41.70
	T3	37.44	0.98	35.50–39.38

Abbreviations: EMM, estimated marginal mean; SE, standard error; CI, confidence interval.

### Toothpaste-by-time interaction

**Fig 2:**
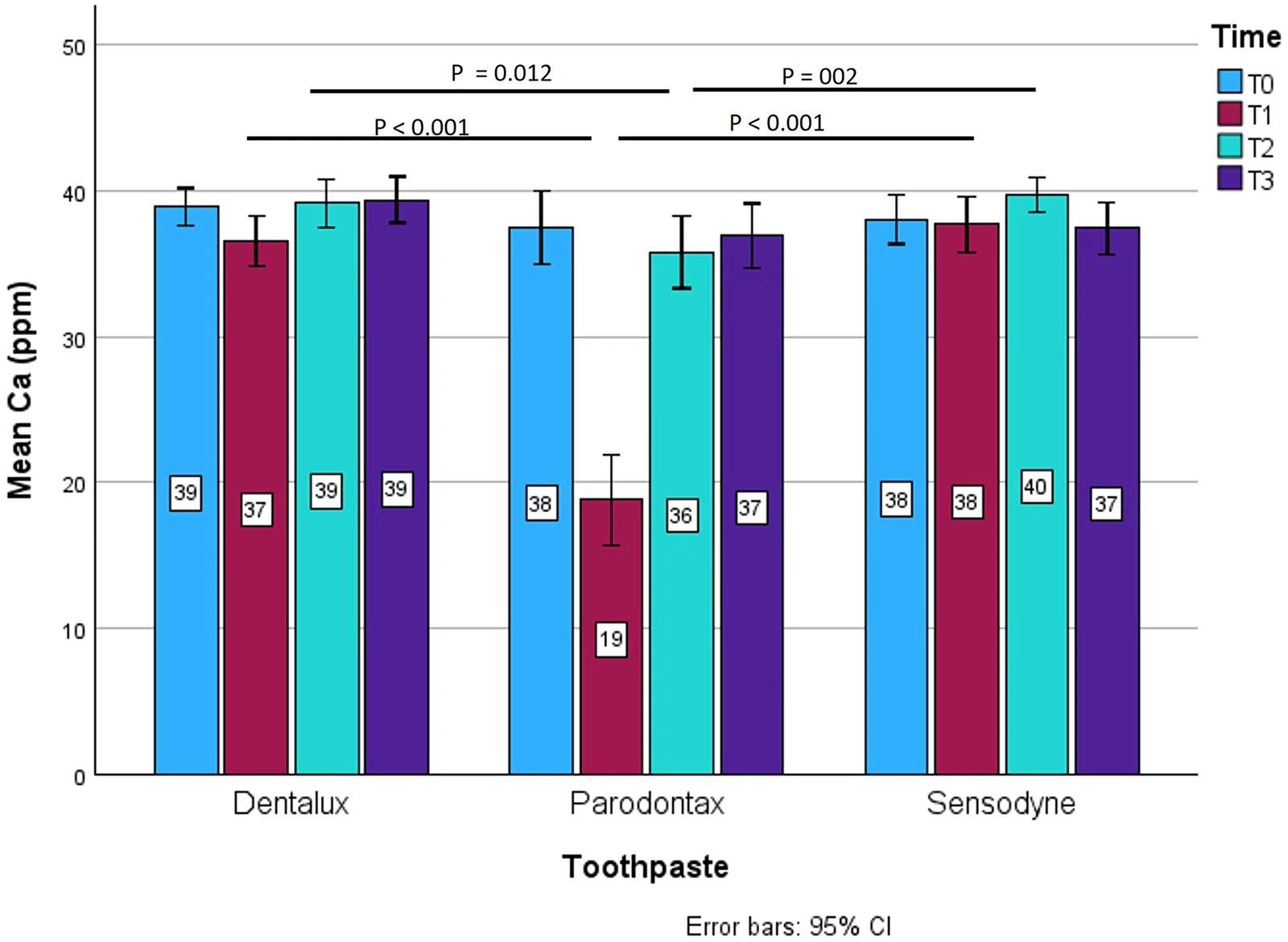
Salivary calcium concentration (ppm) 95% confidence intervals (CIs) according to toothpaste type across four time points (T0–T3). The marked lines indicate statistically significant differences (p ≤ 0.05).

Bonferroni-adjusted pairwise comparisons of the estimated marginal means are summarised in Table 8 and illustrated in Fig 2. At baseline (T0), the salivary calcium concentrations did not differ significantly among the three toothpaste formulations (all Bonferroni-adjusted P ≥ 0.451), confirming comparable pretreatment conditions. Significant between-group differences emerged immediately after toothbrushing (T1). Compared with both Dentalux (mean difference = 17.76 ppm; 95% CI, 13.83–21.70; adjusted P < 0.001) and Sensodyne (mean difference = 18.89 ppm; 95% CI, 14.95–22.83; adjusted P < 0.001), Parodontax produced significantly lower salivary calcium concentrations. No significant difference was observed between Dentalux and Sensodyne (adjusted P = 1.000). At 30 min (T2), salivary calcium concentrations remained significantly in the Parodontax group than the Dentalux group (mean difference = 3.33 ppm; 95% CI, 0.59–6.07; adjusted P = 0.012) and the Sensodyne group (mean difference = 3.92 ppm; 95% CI, 1.18–6.66; adjusted P = 0.002), although these differences were markedly smaller than those observed immediately after brushing. By 60 min (T3), no statistically significant differences in salivary calcium concentrations were detected among the three toothpaste formulations (all adjusted P ≥ 0.081).

**Table 8:** Bonferroni-adjusted pairwise comparisons of estimated marginal means (EMMs) for salivary calcium concentration (ppm) between toothpaste formulations at each measurement time point.

Time	Comparison	Mean difference (ppm)	95% CI	Adjusted P value
T0	Dentalux vs. Parodontax	1.40	−0.95 to 3.74	0.451
	Dentalux vs. Sensodyne	0.86	−1.49 to 3.20	1.000
	Parodontax vs. Sensodyne	−0.54	−2.89 to 1.81	1.000
T1	Dentalux vs. Parodontax	**17.76**	**13.83 to 21.70**	**<0.001**
	Dentalux vs. Sensodyne	−1.13	−5.07 to 2.81	1.000
	Parodontax vs. Sensodyne	**−18.89**	**−22.83 to −14.95**	**<0.001**
T2	Dentalux vs. Parodontax	**3.33**	**0.59 to 6.07**	**0.012**
	Dentalux vs. Sensodyne	−0.59	−3.33 to 2.15	1.000
	Parodontax vs. Sensodyne	**−3.92**	**−6.66 to −1.18**	**0.002**
T3	Dentalux vs. Parodontax	2.50	−0.21 to 5.20	0.081
	Dentalux vs. Sensodyne	1.97	−0.74 to 4.67	0.238
	Parodontax vs. Sensodyne	−0.53	−3.23 to 2.18	1.000

Abbreviations: EMM, estimated marginal mean; CI, confidence interval. The mean differences are expressed as estimated marginal mean differences (ppm). Bonferroni-adjusted P values < 0.05 were considered statistically significant.

### Covariance structure

The estimated covariance parameters are shown in S1 Table. The random intercept variance indicated substantial between-subject variability (variance = 16.44; Wald Z = 3.72; P < 0.001). Furthermore, the covariance parameter estimates confirmed within-subject correlation across repeated measurements, supporting the choice of an unstructured covariance matrix for the linear mixed-effects model.

## Discussion

The primary endpoint of this study was the interaction between toothpaste formulation and measurement time. The highly significant toothpaste×time interaction indicates that changes in salivary calcium concentration after toothbrushing were formulation dependent rather than constant over time. These findings underscore the importance of evaluating temporal responses rather than relying solely on comparisons at individual time points. Among the three tested toothpastes, Parodontax produced the most pronounced short-term response. Immediately after toothbrushing (T1), the salivary calcium concentrations were significantly lower than those observed in the Dentalux and Sensodyne groups, whereas these differences gradually diminished and were no longer statistically significant after 60 min (T3). In contrast, Dentalux and Sensodyne showed relatively stable calcium concentrations throughout the observation period. These results demonstrate that commercially available toothpaste formulations differ in their short-term effects on total salivary calcium concentration after toothbrushing.

The present study was designed as an exploratory comparison of commercially available toothpaste formulations rather than an investigation of individual ingredients. Because the formulations differed in terms of multiple components, including abrasives, surfactants, humectants, flavouring agents, preservatives, and other excipients, the observed effects cannot be attributed to any specific constituents such as sodium bicarbonate, pyrophosphate, zinc, or PVM/MA copolymer. Mechanistic conclusions, therefore, require studies that use otherwise identical experimental formulations but differ in only a single ingredient. Although previous studies have focused primarily on the effects of pyrophosphate on dental calculus formation rather than on salivary calcium concentrations [6–10], the present study extends these observations by demonstrating formulation-dependent differences in short-term changes in total salivary calcium concentration after toothbrushing. For example, salivary calcium concentrations tended to increase between T1 and T3 following the use of Dentalux, whereas Parodontax produced an immediate reduction after toothbrushing. Since only total salivary calcium concentrations were measured, it remains unclear whether these differences reflect changes in biologically available calcium or other formulation-specific mechanisms. Further mechanistic studies are therefore needed to clarify the underlying physiological processes involved.

The randomized crossover design represents an important methodological strength because each participant served as his or her own control, thereby minimizing inter-individual variability [25]. Baseline salivary calcium concentrations were comparable across all treatment periods, supporting the internal validity of the study. Furthermore, the absence of a significant treatment-sequence effect suggests that the order of toothpaste administration did not influence the observed outcomes. A 2-day washout period, randomized treatment allocation, and standardized study procedures were implemented to minimize potential carry-over effects. Although formal estimation of carry-over effects is limited in three-period crossover designs, the comparable baseline values and sensitivity analyses suggest that clinically relevant carry-over effects were unlikely [25,26].

The baseline calcium concentrations observed in this study were consistent with reported physiological concentrations in unstimulated whole saliva, supporting the validity of the measurements [27]. The 60-min observation period was selected in accordance with previous studies investigating short-term changes in salivary ion concentrations [28]. Various analytical methods, including calcium-selective electrodes, have been used to determine salivary calcium concentrations [28,29]. To our knowledge, this is the first randomized crossover study to assess salivary calcium using a colorimetric calcium assay kit in combination with a microplate reader. This approach is well-suited for standardized batch analysis of multiple samples collected at repeated time points and provides reproducible quantification of total salivary calcium [30]. Although calcium-selective electrodes permit direct measurement of ionized calcium, they are less practical for studies involving large numbers of samples [31]. Moreover, previous studies have demonstrated a strong correlation between total salivary calcium concentrations and calcium concentrations in dental biofilm fluid, suggesting that total salivary calcium provides physiologically relevant information despite not reflecting the biologically active ionized fraction [32].

Water rinsing after toothbrushing was intentionally omitted because previous studies have shown that rinsing can influence subsequent salivary calcium measurements [28,29,33]. Consequently, the present protocol allowed assessment of the immediate effects of toothbrushing under standardised conditions. The transient reduction in salivary calcium observed after the use of the Parodontax formulation and the tendency towards increasing calcium concentrations following the use of the Dentalux formulation indicate that different commercially available toothpaste formulations may influence short-term salivary calcium dynamics. However, because the formulations differed in terms of several active and inactive ingredients and because the salivary pH, calcium speciation, phosphate concentration, fluoride concentration, buffering capacity, and salivary flow rate were not assessed, the underlying mechanisms remain uncertain [27,34–36].. Consequently, the observed differences cannot be attributed to individual ingredients or specific physicochemical processes. Future studies combining comprehensive salivary analyses with experimental formulations that differ by only a single ingredient are needed to elucidate the mechanisms responsible for these formulation-dependent effects.

The relatively stable salivary calcium concentrations observed after the use of the Sensodyne formulation may similarly reflect formulation-specific properties rather than the effects of any individual ingredient. Previous in vitro and in situ studies have demonstrated improved fluoride retention and mineral uptake for formulations containing PVM/MA copolymers [13–15]. However, because the present study assessed only total salivary calcium concentrations, no conclusions can be drawn regarding the contribution of this or any other individual ingredient to the observed response.

Several limitations should be considered when interpreting the present findings.

First, only total salivary calcium concentrations were measured in this study Since total calcium does not distinguish between biologically active ionized calcium and other calcium fractions, the results cannot be directly interpreted in terms of calcium bioavailability or remineralization potential. In addition, the salivary flow rate was not assessed and may have influenced the salivary calcium concentration. Future studies should therefore include simultaneous measurements of ionized calcium, phosphate, fluoride, salivary pH, buffering capacity, and salivary flow rate to provide a more comprehensive assessment of salivary mineral homeostasis [37]. Second, the study compared commercially available toothpaste formulations rather than isolated active ingredients. As the formulations differed in terms of multiple components, the observed effects should be interpreted as formulation specific and cannot be attributed to individual ingredients such as pyrophosphate, zinc, sodium bicarbonate, or the PVM/MA copolymer. Experimental studies using otherwise identical formulations differing in only a single component are needed to establish causal relationships. Third, the study included only healthy young adults who were recruited from a single university. Although this homogeneous cohort strengthened the internal validity of the crossover design, the findings may not be generalizable to other populations. Furthermore, only the short-term response following a single toothbrushing episode has been evaluated; the long-term effects on salivary mineral homeostasis, enamel remineralization, dental calculus formation, and caries incidence remain unknown. Finally, the trial was retrospectively registered because it was originally designed as an exploratory physiological study with laboratory-based outcomes rather than as a clinical efficacy trial. Although retrospective registration enhances transparency, it provides limited prespecification of study procedures and outcomes compared with prospective registration and should therefore be considered when interpreting the findings.

Despite these limitations, the study has several notable strengths. To our knowledge, this is the first randomized crossover study to compare the short-term effects of three commercially available fluoride toothpaste formulations on salivary calcium concentrations using repeated measurements obtained before brushing and up to 60 min thereafter. The crossover design minimized interindividual variability, complete follow-up resulted in the absence of missing outcome data, and linear mixed-effects modelling appropriately accounted for the repeated-measures structure of the data. Together, these methodological characteristics provide a robust basis for evaluating short-term formulation-dependent changes in salivary calcium concentration and may inform future mechanistic and clinical investigations into the relationship between toothpaste composition and oral mineral homeostasis.

## Conclusion

This randomized three-treatment, three-period crossover study demonstrated that commercially available fluoride toothpaste formulations differ in their short-term effects on salivary calcium concentration. A significant toothpaste-by-time interaction indicated that the temporal changes in salivary calcium depended on the formulation used. Although baseline calcium concentrations were comparable among the three toothpaste formulations, significant differences were observed immediately after toothbrushing. These differences diminished progressively during the following hour and were no longer statistically significant after 60 min, indicating that the observed effects were transient rather than sustained. Since the investigated products differed in terms of multiple formulation components, the findings should be interpreted as formulation-specific effects rather than evidence of ingredient-specific mechanisms. Furthermore, only total salivary calcium was assessed, and no conclusions can be drawn regarding ionized calcium, enamel remineralization, dental calculus formation, or clinical caries outcomes.

The results of the present study reveal that commercially available fluoride toothpaste formulations can differentially influence short-term salivary calcium dynamics under standardized clinical conditions. Future studies should combine measurements of total and ionized calcium with assessments of salivary pH, phosphate concentration, fluoride concentration, buffering capacity, and salivary flow rate and should evaluate whether these transient biochemical changes translate to clinically relevant effects on remineralization, calculus formation, and caries prevention during long-term toothpaste use.

### Declaration of generative AI and AI-assisted technologies in the writing process

During the preparation of this work, the authors used ChatGPT-4o to improve readability and language. After using this tool, the authors reviewed and edited the content as needed and take full responsibility for the content of the publication.

## Supporting information

S1 File Data for all salivary calcium measurements. (XLSX)

S1 Table Estimated covariance parameters of the linear mixed-effects model, including the random intercept variance and residual covariance structure. (PDF)

## References

[1] Joosstens M, Valkenburg C, Van der Weijden F. Chemical agents to control biofilm formation in step 1 of care-toothpastes and mouthwashes/concepts and challenges. Periodontol 2000. 2025;:10.1111/prd.70022. doi: 10.1111/prd.70022 41277763

[2] Jin Y, Yip H-K. Supragingival calculus: formation and control. Crit Rev Oral Biol Med. 2002;13(5):426–41. doi: 10.1177/154411130201300506 12393761

[3] Vacca Smith AM, Bowen WH. In situ studies of pellicle formation on hydroxyapatite discs. Arch Oral Biol. 2000;45(4):277–91. doi: 10.1016/s0003-9969(99)00141-7 10708668

[4] Hay DI. The interaction of human parotid salivary proteins with hydroxyapatite. Arch Oral Biol. 1973;18(12):1517–29. doi: 10.1016/0003-9969(73)90127-1 4522815

[5] Singh S, Sharma A, Sood PB, Sood A, Zaidi I, Sinha A. Saliva as a prediction tool for dental caries: an in vivo study. J Oral Biol Craniofac Res. 2015;5(2):59–64. doi: 10.1016/j.jobcr.2015.05.001 26258015 PMC4523584

[6] Fairbrother KJ, Kowolik MJ, Curzon ME, Müller I, McKeown S, Hill CM, et al. The comparative clinical efficacy of pyrophosphate/triclosan, copolymer/triclosan and zinc citrate/triclosan dentifrices for the reduction of supragingival calculus formation. J Clin Dent. 1997;8(2 Spec No):62–6. 9238875

[7] Allen DR, Battista GW, Petrone DM, Petrone ME, DeVizio W, Volpe AR. A clinical study to compare the anticalculus efficacy of three dentifrice formulations. J Clin Dent. 2002;13(2):69–72. 11695209

[8] Bollmer BW, Sturzenberger OP, Vick V, Grossman E. Reduction of calculus and Peridex stain with Tartar-Control Crest. J Clin Dent. 1995;6(4):185–7. 8624229

[9] Pradeep AR, Agarwal E, P AR, Rao MSN, Faizuddin M. Study of orthophosphate, pyrophosphate, and pyrophosphatase in saliva with reference to calculus formation and inhibition. J Periodontol. 2011;82(3):445–51. doi: 10.1902/jop.2010.100355 20843234

[10] Cvjetinovic A, Ramseier CA, Salvi GE, Laugisch O. Chemical additives in toothpastes to inhibit calculus formation. Swiss Dent J. 2020;130(6):503–13. doi: 10.61872/sdj-2020-06-03 32512986

[11] Ingram GS, Horay CP, Stead WJ. Interaction of zinc with dental mineral. Caries Res. 1992;26(4):248–53. doi: 10.1159/000261447 1330308

[12] Adams D. Calculus-inhibition agents: a review of recent clinical trials. Adv Dent Res. 1995;9:410–8.

[13] Kim CS, Ozer F, Mante FK. Mechanics of dental adhesives supplemented with polymethyl-vinyl-ether-co-maleic anhydride. J Adhes Sci Technol. 2017;31:1116–24. doi: 10.1080/01694243.2016.1245569

[14] Gaffar A, Esposito A, Afflitto J. In vitro and in vivo anticalculus effects of a triclosan/copolymer system. Am J Dent. 1990;3 Spec No:S37–42. 1964561

[15] Hattab FN. Remineralisation of carious lesions and fluoride uptake by enamel exposed to various fluoride dentifrices in vitro. Oral Health Prev Dent. 2013;11(3):281–90. doi: 10.3290/j.ohpd.a30170 23878839

[16] Mellberg JR, Petrou ID, Fletcher R, Grote N. Evaluation of the effects of a pyrophosphate-fluoride anticalculus dentifrice on remineralization and fluoride uptake in situ. Caries Res. 1991;25(1):65–9. doi: 10.1159/000261344 1649005

[17] Sullivan RJ, Fletcher R, Bachiman R, Penugonda B, LeGeros RZ. Intra-oral comparison and evaluation of the ability of fluoride dentifrices to promote the remineralization of caries-like lesions in dentin and enamel. J Clin Dent. 1995;6(2):135–8. 8624224

[18] Tanzer JM, Pellegrino J, Thompson AM, Buch RM. Verification of caries inhibition by a tartar control toothpaste. J Clin Dent. 2003;14(3):74–6. 14520778

[19] Ripa LW, Leske GS, Triol CW, Volpe AR. Clinical study of the anticaries efficacy of three fluoride dentifrices containing anticalculus ingredients: three-year (final) results. J Clin Dent. 1990;2(2):29–33. 1965288

[20] Beiswanger BB, Gish CW, Mallatt ME. A three-year study of the effect of a sodium fluoride-silica abrasive dentifrice on dental caries. Pharmacol Ther Dent. 1981;6(1–2):9–16. 6264507

[21] Zacherl WA. A three-year clinical caries evaluation of the effect of a sodium fluoride-silica abrasive dentifrice. Pharmacol Ther Dent. 1981;6(1–2):1–7. 6264506

[22] Duckworth RM, Huntington E. Evidence for putting the calculus: caries inverse relationship to work. Community Dent Oral Epidemiol. 2005;33(5):349–56. doi: 10.1111/j.1600-0528.2005.00227.x 16128794

[23] Levine RS. Pyrophosphates in toothpaste: a retrospective and reappraisal. Br Dent J. 2020;229(10):687–9. doi: 10.1038/s41415-020-2346-4 33247264

[24] Faul F, Erdfelder E, Buchner A, Lang A-G. Statistical power analyses using G*Power 3.1: tests for correlation and regression analyses. Behav Res Methods. 2009;41(4):1149–60. doi: 10.3758/BRM.41.4.1149 19897823

[25] Antczak-Bouckoms AA, Tulloch JF, Berkey CS. Split-mouth and cross-over designs in dental research. J Clin Periodontol. 1990;17(7 Pt 1):446–53. doi: 10.1111/j.1600-051x.1990.tb02343.x 2201705

[26] Lim C-Y, In J. Considerations for crossover design in clinical study. Korean J Anesthesiol. 2021;74(4):293–9. doi: 10.4097/kja.21165 34344139 PMC8342834

[27] Larsen MJ, Pearce EIF. Saturation of human saliva with respect to calcium salts. Arch Oral Biol. 2003;48(4):317–22. doi: 10.1016/s0003-9969(03)00007-4 12663077

[28] Parkinson CR, Burnett GR, Thomas GV, Davies L, Payne D. Randomised study of intra-oral kinetics of fluoride-containing toothpastes. J Dent. 2021;106:103587. doi: 10.1016/j.jdent.2021.103587 33508354

[29] Burnett G, Nehme M, Parkinson C, Karwal R, Badrock T, Thomas GV, et al. A randomised oral fluoride retention study comparing intra-oral kinetics of fluoride-containing dentifrices before and after dietary acid exposure. Arch Oral Biol. 2020;119:104891. doi: 10.1016/j.archoralbio.2020.104891 32937232

[30] Mir AA, Goyal B, Datta SK, Ikkurthi S, Pal A. Comparison between measured and calculated free calcium values at different serum albumin concentrations. J Lab Physicians. 2016;8(2):71–6. doi: 10.4103/0974-2727.180785 27365914 PMC4866387

[31] Carey CM, Vogel GL. Measurement of calcium activity in oral fluids by ion selective electrode: method evaluation and simplified calculation of ion activity products. J Res Natl Inst Stand Technol. 2000;105(2):267–73. doi: 10.6028/jres.105.030 27551609 PMC4872681

[32] Matsuo S, Lagerlöf F. Relationship between total and ionized calcium concentrations in human whole saliva and dental plaque fluid. Arch Oral Biol. 1991;36(7):525–7. doi: 10.1016/0003-9969(91)90146-l 1776927

[33] Vogel GL, Zhang Z, Chow LC, Schumacher GE. Effect of a water rinse on “labile” fluoride and other ions in plaque and saliva before and after conventional and experimental fluoride rinses. Caries Res. 2001;35(2):116–24. doi: 10.1159/000047442 11275671

[34] Madeswaran S, Jayachandran S. Sodium bicarbonate: a review and its uses in dentistry. Indian J Dent Res. 2018;29(5):672–7. doi: 10.4103/ijdr.IJDR_30_17 30409952

[35] Dawes C. Salivary flow patterns and the health of hard and soft oral tissues. J Am Dent Assoc. 2008;139 Suppl:18S-24S. doi: 10.14219/jada.archive.2008.0351 18460676

[36] Wang W, Xie Q, Xu T, Wang Q, Malmstrom HS, Ren Y-F. Fluoride release and anti-erosive effects of dentifrices containing PVM/MA copolymers. J Dent. 2013;41(2):148–54. doi: 10.1016/j.jdent.2012.10.013 23123494

[37] Dawes C, Wong DTW. Role of saliva and salivary diagnostics in the advancement of oral health. J Dent Res. 2019;98(2):133–41. doi: 10.1177/0022034518816961 30782091 PMC6900436

