# Supplementary material for: Short-term effects of different toothpaste formulations on total salivary calcium: A randomized crossover study
Source: PLoS One. 2026 Sep 17;21(9):e0358715. doi: 10.1371/journal.pone.0358715 (PMC13585233; doi:10.1371/journal.pone.0358715)
Supplement: Supplementary file 1 [file pone.0358715.s002.pdf]

Covariance structure

S3 Table. Estimated covariance parameters of the linear mixed-effects model, including the random intercept variance and residual covariance structure.

Covariance parameter	Estimate	Wald Z	P value	95% CI
**Random intercept variance (participant)**	16.44	3.72	<0.001	9.70–27.85
**Residual variance (T0)**	22.18	6.68	<0.001	16.54–29.74
**Residual variance (T1)**	63.04	7.66	<0.001	48.80–81.43
**Residual variance (T2)**	30.64	7.75	<0.001	23.79–39.45
**Residual variance (T3)**	29.67	7.42	<0.001	22.78–38.63
