# Supplementary material for: Short-term effects of different toothpaste formulations on total salivary calcium: A randomized crossover study
Source: PLoS One. 2026 Sep 17;21(9):e0358715. doi: 10.1371/journal.pone.0358715 (PMC13585233; doi:10.1371/journal.pone.0358715)
Supplement: Supplementary file 2 [file pone.0358715.s004.docx]

**Response to the Academic Editor**

We sincerely thank the Academic Editor for the careful evaluation of our manuscript and for the constructive and detailed comments. We appreciate the time and effort invested in reviewing our work. We have carefully considered all comments raised by the Academic Editor and the reviewers and have substantially revised the manuscript accordingly. These revisions have improved the clarity, methodological transparency, statistical reporting, and overall quality of the manuscript. All changes are highlighted in the revised manuscript with tracked changes. A detailed point-by-point response is provided below.

**Comment 1:** *Although Reviewer 3 raised only minor comments, both Reviewers 1 and 2 identified substantial methodological and reporting deficiencies, particularly regarding the description of the cross-over design, adherence to CONSORT recommendations, statistical analysis, and interpretation of the findings.*

**Response:**
We appreciate this important comment and have comprehensively revised the manuscript. The description of the randomized three-treatment, three-period crossover design has been substantially expanded in the Methods section. The study design, randomization procedure, treatment sequences, washout period, and statistical analysis are now described in considerably greater detail. Furthermore, the manuscript has been revised to improve adherence to the CONSORT 2010 statement and the CONSORT extension for randomized crossover trials where applicable. The statistical analysis was restructured using a linear mixed-effects model with participant as a random effect and toothpaste formulation, measurement time, treatment sequence, and the toothpaste-by-time interaction as fixed effects. The Results and Discussion sections were rewritten to reflect these analyses, and the interpretation of the findings has been substantially moderated to avoid conclusions that extend beyond the presented data.

**Comment 2:** *The Introduction is excessively long.*

**Response:**
The Introduction has been substantially shortened and reorganized. Background information that was not directly relevant to the study objectives was removed, while the rationale for investigating short-term changes in salivary calcium concentration following the use of different toothpaste formulations was strengthened. Redundant descriptions of pellicle formation, saliva composition, pyrophosphate chemistry, and previous literature were condensed to improve readability and focus.

**Comment 3:** *Insufficient justification for recruiting only university students.*

**Response:**
Additional justification has been included in the Methods and Discussion sections. Healthy university students were selected to obtain a relatively homogeneous study population with comparable oral and general health conditions, thereby minimizing biological variability in this exploratory crossover study. We now explicitly acknowledge that this recruitment strategy may limit the generalizability of the findings and identify this as a limitation of the study.

**Comment 4:** *Inadequate reporting of trial registration.*

**Response:**
We have revised the manuscript to clarify the timing of trial registration. Because the study was originally designed as an exploratory physiological crossover investigation with laboratory analysis of saliva as the primary outcome and without evaluation of clinical efficacy endpoints, prospective trial registration was not initially considered. Following the editor's recommendation, the study has now been retrospectively registered, and the registration number has been added to the manuscript. We explicitly acknowledge the retrospective registration and discuss this transparently in the Methods section in accordance with PLOS ONE reporting requirements.

**Comment 5:** *Limited description of salivary flow assessment.*

**Response:**
The Methods section has been expanded to provide a detailed description of saliva collection. We now clarify that unstimulated whole saliva was collected under standardized conditions at all four measurement time points. We further explain that salivary flow rate was not measured because the primary objective was the determination of calcium concentration rather than total calcium secretion. This limitation has been explicitly acknowledged in the Discussion, where we recommend inclusion of salivary flow measurements in future studies.

**Comment 6:** *Numerous unsupported statements in the Introduction and Discussion.*

**Response:**
The Introduction and Discussion have been carefully revised to remove unsupported or overly speculative statements. Several sections were rewritten to distinguish clearly between established evidence from the literature and interpretations derived from the present findings. Additional references were incorporated where appropriate, while speculative mechanistic explanations are now explicitly identified as hypotheses requiring further investigation.

**Comment 7:** *Table 2 is misleading and does not accurately represent the crossover study design.*

**Response:**
We fully agree with this comment. The original figure and accompanying table have been redesigned to clearly illustrate the three-treatment, three-period crossover design. The revised study flow diagram now demonstrates that all 48 participants received each of the three toothpaste formulations according to one of the randomized treatment sequences (ABC, BCA, or CAB), separated by standardized 48-hour washout periods. The revised figure also illustrates participant progression through all treatment periods and saliva collection time points (T0–T3), thereby avoiding the previous impression of three independent parallel groups.

**Response to Reviewer #1**

We sincerely thank Reviewer #1 for the thorough and constructive evaluation of our manuscript. We appreciate the positive assessment of the scientific relevance of the study and the many valuable suggestions for improving the methodological transparency and statistical analysis. We have carefully considered every comment and have substantially revised the manuscript accordingly. Our detailed responses are provided below.

**General comment**

*The manuscript presents analysis of data generated from a randomized cross-over clinical study evaluating the short-term effects of three commercially available toothpaste formulations on total salivary calcium concentrations in healthy young adults. The topic is relevant, the study is generally clearly written, the experimental procedures are described in detail...*

**Response:**

We sincerely thank the reviewer for the positive evaluation of the study and for recognizing the relevance of our research question. We appreciate the constructive suggestions, which have substantially improved the clarity, methodological rigor, and statistical reporting of the manuscript.

**Comment 1**

*Methods reporting needs improvement. The manuscript should follow the CONSORT guidelines and use separate subsections such as Trial design, Participants, Interventions, Outcomes, Sample size, Randomization, Blinding, Statistical analysis, etc.*

**Response:**

We agree with this important recommendation. The Methods section has been completely reorganized following the CONSORT 2010 Statement and the CONSORT extension for randomized crossover trials where applicable. Separate subsections have been introduced for Ethics, Sample Size, study design, participant recruitment, Randomization, interventions, outcome measures; saliva collection, laboratory analysis, and statistical analysis. Redundant information has been removed, and the methodological description has been substantially expanded to improve transparency and reproducibility.

**Comment 2a**

*Randomization and allocation concealment should be described separately.*

**Response:**

Thank you for this suggestion. The manuscript now clearly distinguishes between sequence randomization and allocation concealment. The randomized treatment sequences were generated before participant recruitment by an independent investigator who was not involved in enrolment or outcome assessment. Allocation was concealed until participant assignment, and investigators performing the laboratory analyses remained blinded to treatment allocation until completion of data analysis.

“Randomization. Participants were assigned to the treatment sequences using a computer-generated randomization list with a 1:1 allocation ratio. The randomization sequence was generated prior to participant enrollment by an investigator who was not involved in participant recruitment or outcome assessment.”

“Allocation concealment. Allocation concealment was ensured by using sequentially numbered, opaque, sealed envelopes containing the treatment assignments. The envelopes were opened only after a participant had been enrolled, thereby preventing foreknowledge of the assigned treatment sequence.”

**Comment 2b**

*More details on the randomization procedure are needed.*

**Response:**

Additional details have been incorporated into the Methods section. The revised manuscript now describes the three-sequence Latin-square crossover design (ABC, BCA, and CAB), the generation of the randomization list, participant allocation, and the implementation of the randomized treatment sequences.

**Comment 3a**

*Repeated-measures ANOVA does not adequately account for sequence, period, and carry-over effects.*

**Response:**

We fully agree and thank the reviewer for this important observation. Following this recommendation, the statistical analysis has been completely revised. The previous repeated-measures ANOVA has been replaced by a linear mixed-effects model estimated by restricted maximum likelihood (REML). Toothpaste formulation, measurement time, treatment sequence, and the toothpaste-by-time interaction were included as fixed effects, whereas participant was included as a random intercept. Treatment sequence was retained in the final model to account for possible sequence-related effects. Because of the characteristics of the three-sequence Latin-square design, period and sequence were collinear and could not be entered simultaneously into the same model. This issue is now explicitly explained in the Methods section, and sensitivity analyses showed no evidence of clinically meaningful carry-over effects.

**Comment 3b**

*A linear mixed-effects model would have been more appropriate.*

**Response:**

We appreciate this recommendation and have implemented it in the revised manuscript. The entire statistical analysis has been repeated using linear mixed-effects modelling. The Results section has been rewritten accordingly and now includes fixed-effect parameter estimates, Type III tests of fixed effects, estimated marginal means with 95% confidence intervals, Bonferroni-adjusted pairwise comparisons, and covariance parameter estimates.

**Comment 3c**

*Model assumptions were not assessed.*

**Response:**

Thank you for highlighting this point. Model assumptions were evaluated by visual inspection of residual histograms, normal Q–Q plots, and residual-versus-fitted plots. No substantial deviations from normality or homoscedasticity were observed. This information has been added to the Results section.

**Comment 3d**

*Independent-samples tests are inappropriate for a crossover design.*

**Response:**

We agree. Independent-samples analyses have been removed from the manuscript. All statistical analyses are now based on the linear mixed-effects model, which appropriately accounts for repeated observations within participants and the crossover study design. Pairwise comparisons were performed using estimated marginal means derived from the fitted model with Bonferroni adjustment for multiple testing.

**Comment 3e**

*Confidence intervals should be incorporated into the interpretation.*

**Response:**

We appreciate this suggestion. Throughout the revised Results and Discussion, treatment effects are now interpreted using both effect estimates and their corresponding 95% confidence intervals rather than relying solely on P values. This revision provides a more balanced assessment of the magnitude and precision of the observed effects and is consistent with current recommendations for reporting clinical studies.

**Comment 4a**

*Statements of significance should always be accompanied by P values.*

**Response:**

The Results section has been carefully revised. All statements referring to statistically significant findings are now accompanied by the corresponding P values.

**Comment 4b**

*The conclusions should acknowledge that the study population consisted only of participants from Witten/Herdecke University.*

**Response:**

We agree and have revised both the Discussion and the Conclusion accordingly. We now explicitly state that the study population consisted exclusively of healthy volunteers recruited from Witten/Herdecke University and acknowledge that this may limit the generalizability of the findings. We further emphasize that larger multicentre studies including more diverse populations are required to confirm the present results.

**Response to Reviewer #2**

We sincerely thank Reviewer #2 for the thorough evaluation of our manuscript and for the many constructive comments and suggestions. We appreciate the detailed feedback, which has substantially improved the quality, clarity, and scientific rigor of our manuscript. Each comment has been carefully addressed below, and all corresponding changes have been incorporated into the revised manuscript.

**Response to Reviewer #3**

We sincerely thank Reviewer #3 for the careful review of our manuscript and for the constructive suggestions. We appreciate the positive evaluation of our work and have revised the manuscript accordingly. All comments have been addressed individually below, and the corresponding revisions have been incorporated into the manuscript.
