# Supplementary material for: Short-term effects of different toothpaste formulations on total salivary calcium: A randomized crossover study
Source: PLoS One. 2026 Sep 17;21(9):e0358715. doi: 10.1371/journal.pone.0358715 (PMC13585233; doi:10.1371/journal.pone.0358715)
Supplement: Peer review history

# Peer review: Decision Letter 0

**DOI:** 10.1371/journal.pone.0358715.r001

**Authors:** Esra Cengiz Yanardag

30 Jun 2026

Dear Dr. Aminy,

Thank you for submitting your manuscript to PLOS ONE. After careful consideration, we feel that it has merit but does not fully meet PLOS ONE’s publication criteria as it currently stands. Therefore, we invite you to submit a revised version of the manuscript that addresses the points raised during the review process.

==============================

**ACADEMIC EDITOR:**

==============================

Please submit your revised manuscript by Aug 14 2026 11:59PM. If you will need more time than this to complete your revisions, please reply to this message or contact the journal office at plosone@plos.org. When you're ready to submit your revision, log on to https://www.editorialmanager.com/pone/ and select the 'Submissions Needing Revision' folder to locate your manuscript file.

- A letter that responds to each point raised by the academic editor and reviewer(s). You should upload this letter as a separate file labeled 'Response to Reviewers'.
- A marked-up copy of your manuscript that highlights changes made to the original version. You should upload this as a separate file labeled 'Revised Manuscript with Track Changes'.
- An unmarked version of your revised paper without tracked changes. You should upload this as a separate file labeled 'Manuscript'.

If you would like to make changes to your financial disclosure, please include your updated statement in your cover letter. Guidelines for resubmitting your figure files are available below the reviewer comments at the end of this letter.

If applicable, we recommend that you deposit your laboratory protocols in protocols.io to enhance the reproducibility of your results. Protocols.io assigns your protocol its own identifier (DOI) so that it can be cited independently in the future. For instructions see: https://journals.plos.org/plosone/s/submission-guidelines#loc-laboratory-protocols. Additionally, PLOS ONE offers an option for publishing peer-reviewed Lab Protocol articles, which describe protocols hosted on protocols.io. Read more information on sharing protocols at https://plos.org/protocols?utm_medium=editorial-email&utm_source=authorletters&utm_campaign=protocols.

As the corresponding author, your ORCID iD is verified in the submission system and will appear in the published article. PLOS supports the use of ORCID, and we encourage all coauthors to register for an ORCID iD and use it as well. Please encourage your coauthors to verify their ORCID iD within the submission system before final acceptance, as unverified ORCID iDs will not appear in the published article. *Only* the individual author can complete the verification step; PLOS staff *cannot* verify ORCID iDs on behalf of authors.

We look forward to receiving your revised manuscript.

Kind regards,

Esra Cengiz Yanardag

Academic Editor

PLOS One

Journal requirements:

When submitting your revision, we need you to address these additional requirements.

1. Please ensure that your manuscript meets PLOS ONE's style requirements, including those for file naming. The PLOS ONE style templates can be found at

https://journals.plos.org/plosone/s/file?id=wjVg/PLOSOne_formatting_sample_main_body.pdf and

https://journals.plos.org/plosone/s/file?id=ba62/PLOSOne_formatting_sample_title_authors_affiliations.pdf

2. Thank you for stating the following in the Funding Section of your manuscript:

“This study was supported by the Fördergemeinschaft of Witten/Herdecke University. The funding source had no involvement in the study design; collection, analysis, or interpretation of data; writing of the manuscript; or the decision to submit the article for publication.”

We note that you have provided funding information that is currently declared in your Funding Statement. However, funding information should not appear in the Acknowledgments section or other areas of your manuscript. We will only publish funding information present in the Funding Statement section of the online submission form.

Please remove any funding-related text from the manuscript and let us know how you would like to update your Funding Statement. Currently, your Funding Statement reads as follows:

“This study was supported by the Fördergemeinschaft of Witten/Herdecke University. The funding source had no involvement in the study design; collection, analysis, or interpretation of data; writing of the manuscript; or the decision to submit the article for publication.”

Please include your amended statements within your cover letter; we will change the online submission form on your behalf.

3. Your ethics statement should only appear in the Methods section of your manuscript. If your ethics statement is written in any section besides the Methods, please delete it from any other section.

4. PLOS requires an ORCID iD for the corresponding author in Editorial Manager on papers submitted after December 6th, 2016. Please ensure that you have an ORCID iD and that it is validated in Editorial Manager. To do this, go to ‘Update my Information’ (in the upper left-hand corner of the main menu), and click on the Fetch/Validate link next to the ORCID field. This will take you to the ORCID site and allow you to create a new iD or authenticate a pre-existing iD in Editorial Manager.

If the reviewer comments include a recommendation to cite specific previously published works, please review and evaluate these publications to determine whether they are relevant and should be cited. There is no requirement to cite these works unless the editor has indicated otherwise.

[Note: HTML markup is below. Please do not edit.]

Reviewers' comments:

Reviewer's Responses to Questions

**Comments to the Author**

1. Is the manuscript technically sound, and do the data support the conclusions?

Reviewer #1: Partly

Reviewer #2: No

Reviewer #3: Partly

**********

2. Has the statistical analysis been performed appropriately and rigorously?

Reviewer #1: No

Reviewer #2: No

Reviewer #3: No

**********

3. Have the authors made all data underlying the findings in their manuscript fully available?

The PLOS Data policy

Reviewer #1: Yes

Reviewer #2: Yes

Reviewer #3: Yes

**********

4. Is the manuscript presented in an intelligible fashion and written in standard English?

Reviewer #1: Yes

Reviewer #2: Yes

Reviewer #3: Yes

**********

Reviewer #1: The manuscript presents analysis of data generated from a randomized cross-over clinical study evaluating the short-term effects of three commercially available toothpaste formulations on total salivary calcium concentrations in healthy young adults. The topic is relevant, the study is generally clearly written, the experimental procedures are described in detail, and the cross-over design is appropriate for reducing between-subject variability. While the study objectives sound interesting, is important, and on target, some shortcomings were observed in regards to abiding by the CONSORT guidelines for conducting and reporting results of high-quality randomized controlled trials (RCTs).

1. My first comment is related to the Abstract. It would better serve the readers, if it was rewritten as: Objectives/Methods/Results/Conclusions subsections.

Some other (mostly statistical) comments appear below:

2. Methods:

Methods reporting need some work. An orderly manner is suggested, following CONSORT guidelines, without repeating information, such as Trial Design, Participant Eligibility Criteria and settings, Interventions, Outcomes, sample size/power considerations, Interim analysis and stopping rules, Randomization (details on random number generation, allocation concealment, implementation), Blinding issues, etc, should be mentioned. The authors are advised to create separate subsections for each of the possible topics (whichever necessary), and that way produce a very clear writeup. They are advised to write it carefully, following nice examples in the manuscript below:

https://www.sciencedirect.com/science/article/pii/S0889540619300010

Specific comments:

(a) For instance, the randomization and allocation concealment should be made very clear (they are NOT the same thing); the trial staff recruiting patients should NOT have the randomization list. Randomization should be prepared by the trial statistician, and he/she would not participate in the recruiting.

(b) More details on the randomization is needed; there is minimal information.

(c) Statistical Analysis writeup:

(c1) Although the underlying design is cross-over, the analytical method (repeated-measures ANOVA) do not seem to consider the various nuances of the design, for example, treatment sequence effects, period effects, carry-over effects.

(c2) A linear mixed-effects modeling (LMMs) framework would have been more appropriate, due to the hierarchical nature of the data – repeated time-points nested within treatment periods. LMMs can elegantly accommodate within-subject correlation, missingness issues, treatment X time interactions, etc. Not clear, why it was not done.

(c3) Repeated-measures ANOVA are mostly based on Gaussian assumptions; violations to those assumpstions were not assessed in the data analysis.

(c4) Since the same individuals received all toothpaste formulations in a cross-over design, independent-samples tests are inappropriate; paired analyses are likely required throughout.

(c5) Confidence intervals are reported descriptively but are not meaningfully incorporated into the interpretation of results. Consequently, the discussion remains predominantly p-value driven and does not adequately address the precision or clinical importance of the estimated effects. Some effort was expected.

3. Results & Conclusions:

(a) The authors should check that any statement of significance should be followed by a p-value in the entire Results section. Otherwise, the Results section look OK; it's pretty straightforward.

(b) Conclusions should state that the current findings are ONLY based on the samples derived from the Witten/Herdecke University, and should allude to future studies with much larger sample sizes and collected at other geographical areas to confirm the effectiveness of the competing toothpastes.

Reviewer #2: This manuscript addresses an interesting and potentially relevant question: whether commercially available fluoride toothpastes with different adjunctive agents produce short-term changes in salivary calcium concentration. However, I have substantial concerns regarding the scientific rigor of the study design, the description of the cross-over methodology, and the appropriateness of the statistical analyses.

First, the cross-over design is insufficiently described and appears internally inconsistent. It is not clear whether all participants actually tested all three toothpaste formulations, as would be expected in a true cross-over trial. The manuscript also does not adequately describe the randomization sequence, allocation procedure, period structure, washout rationale, or assessment of possible period and carry-over effects. These omissions make it difficult to determine whether the study was genuinely conducted and analyzed as a cross-over trial.

Second, the statistical methods are inconsistently described and raise methodological concerns. The manuscript refers to repeated-measures ANOVA, paired comparisons, and independent-samples t-tests in ways that are not fully coherent with a cross-over repeated-measures design. If the same participants were exposed to multiple toothpaste formulations, then independent-samples tests would generally be inappropriate. These inconsistencies suggest a limited understanding of the statistical requirements for cross-over clinical studies and undermine confidence in the reported inferences.

Third, although this is presented as a clinical trial, the manuscript does not clearly define a primary endpoint. It is unclear whether the main outcome was the absolute calcium concentration at a specific time point, the change from baseline, the overall time-by-treatment interaction, or another prespecified comparison. Without a clearly defined primary endpoint and primary statistical contrast, the analysis appears exploratory and vulnerable to selective interpretation of multiple post hoc comparisons.

A further major concern is the use of commercially available toothpaste formulations to infer ingredient-specific mechanisms. Because each product differs in multiple components beyond the presumed active adjunctive agent, the observed effects cannot be attributed confidently to pyrophosphate, zinc, sodium bicarbonate, or PVM/MA copolymer alone. In this type of product-level comparison, mechanistic interpretation at the ingredient level is inherently limited unless matched experimental formulations or additional analytical controls are used. The manuscript also does not provide a sufficiently clear rationale for why these particular commercial toothpastes were selected, nor does it adequately justify treating them as representative of the ingredients of interest.

Overall, while the research question is potentially worthwhile, the current manuscript has serious weaknesses in experimental design, methodological reporting, endpoint definition, and statistical implementation. The conclusions should be substantially tempered, and the study should be reframed as an exploratory comparison of three commercial products rather than as evidence for specific ingredient-driven

Reviewer #3: Dear authors,

The manuscript reports a randomized cross-over trial assessing short‑term effects of three fluoride toothpastes (NaF + pyrophosphate + zinc; NaF + sodium bicarbonate; NaF + PVM/MA) on total salivary calcium concentration over 60 minutes. The topic is relevant, the manuscript is generally well written, and ethical aspects, registration, and data availability are addressed.

Minor considerations have to be done.

- Assay description: fluorescence vs colorimetric

The Abstract states (line 37): “Calcium concentrations were determined using a fluorescence‑based Ca²⁺ assay kit.”

The chapter Methods specify (line 205) a colorimetric assay (Abcam ab102505, chromogenic reagent, absorbance at 575 nm).

This is a clear internal inconsistency and could mislead readers. Please correct the Abstract and any other mentions to consistently describe the assay as colorimetric, not fluorescence‑based.

Textual issues and typos:

- “ll individuals” → “All individuals” (Materials and Methods, Line 162).

- from the student of Witten/Herdecke University” → “from the students of Witten/Herdecke University” (line 151).

- Recruitment period and trial registration

- Table 3

(Line 287) What mean Fig 2?

-Salivary flow rate, pH

Salivary flow rates are part of the inclusion criteria but are later mentioned as not investigated.

It would be helpful to:

-briefly describe the method used to determine the stimulated and unstimulated flow rates at screening,

-clarify that flow rates were not measured at each study visit and note this as a limitation (possible influence on calcium concentrations).

The same for the pH. The pH value has an impact on Ca2+-concentration in saliva. Pleasse explain, why this measurement is neglectable in this study ( primary mechanism chelatisation?).

- Units and conversion to ppm

The assay provides sample amounts in µg; results are reported in ppm.

Please briefly explain: The conversion from µg/well to ppm in saliva (including assumptions about sample volume and density). Whether ppm here is equivalent to mg/L (as is common in aqueous solutions).

- Reference details

(line 421) Joosstens et al., Periodontol 2000, 2025). Please verify that these are correct and, if they are “ahead of print”, indicate this clearly or update when final bibliographic details are available like DOI.

- Ex347

What is Ex347? I could not find any information about. Please describe briefly.

With kind regards

The reviewer

**********

what does this mean?). If published, this will include your full peer review and any attached files.

If you choose “no”, your identity will remain anonymous but your review may still be made public.

**Do you want your identity to be public for this peer review?** For information about this choice, including consent withdrawal, please see our Privacy Policy

Reviewer #1: No

Reviewer #2: No

Reviewer #3: No

**********

[NOTE: If reviewer comments were submitted as an attachment file, they will be attached to this email and accessible via the submission site. Please log into your account, locate the manuscript record, and check for the action link "View Attachments". If this link does not appear, there are no attachment files.]

To ensure your figures meet our technical requirements, please review our figure guidelines: https://journals.plos.org/plosone/s/figures

You may also use PLOS’s free figure tool, NAAS, to help you prepare publication quality figures: https://journals.plos.org/plosone/s/figures#loc-tools-for-figure-preparation.

NAAS will assess whether your figures meet our technical requirements by comparing each figure against our figure specifications.

# Author comment: Author response to Decision Letter 1

**DOI:** 10.1371/journal.pone.0358715.r002

17 Jul 2026

Response to the Academic Editor

We sincerely thank the Academic Editor for the careful evaluation of our manuscript and for the constructive and detailed comments. We appreciate the time and effort invested in reviewing our work. We have carefully considered all comments raised by the Academic Editor and the reviewers and have substantially revised the manuscript accordingly. These revisions have improved the clarity, methodological transparency, statistical reporting, and overall quality of the manuscript. All changes are highlighted in the revised manuscript with tracked changes. A detailed point-by-point response is provided below.

Comment 1: Although Reviewer 3 raised only minor comments, both Reviewers 1 and 2 identified substantial methodological and reporting deficiencies, particularly regarding the description of the cross-over design, adherence to CONSORT recommendations, statistical analysis, and interpretation of the findings.

Response:

We appreciate this important comment and have comprehensively revised the manuscript. The description of the randomized three-treatment, three-period crossover design has been substantially expanded in the Methods section. The study design, randomization procedure, treatment sequences, washout period, and statistical analysis are now described in considerably greater detail. Furthermore, the manuscript has been revised to improve adherence to the CONSORT 2010 statement and the CONSORT extension for randomized crossover trials where applicable. The statistical analysis was restructured using a linear mixed-effects model with participant as a random effect and toothpaste formulation, measurement time, treatment sequence, and the toothpaste-by-time interaction as fixed effects. The Results and Discussion sections were rewritten to reflect these analyses, and the interpretation of the findings has been substantially moderated to avoid conclusions that extend beyond the presented data.

Comment 2: The Introduction is excessively long.

Response:

The Introduction has been substantially shortened and reorganized. Background information that was not directly relevant to the study objectives was removed, while the rationale for investigating short-term changes in salivary calcium concentration following the use of different toothpaste formulations was strengthened. Redundant descriptions of pellicle formation, saliva composition, pyrophosphate chemistry, and previous literature were condensed to improve readability and focus.

Comment 3: Insufficient justification for recruiting only university students.

Response:

Additional justification has been included in the Methods and Discussion sections. Healthy university students were selected to obtain a relatively homogeneous study population with comparable oral and general health conditions, thereby minimizing biological variability in this exploratory crossover study. We now explicitly acknowledge that this recruitment strategy may limit the generalizability of the findings and identify this as a limitation of the study.

Comment 4: Inadequate reporting of trial registration.

Response:

We have revised the manuscript to clarify the timing of trial registration. Because the study was originally designed as an exploratory physiological crossover investigation with laboratory analysis of saliva as the primary outcome and without evaluation of clinical efficacy endpoints, prospective trial registration was not initially considered. Following the editor's recommendation, the study has now been retrospectively registered, and the registration number has been added to the manuscript. We explicitly acknowledge the retrospective registration and discuss this transparently in the Methods section in accordance with PLOS ONE reporting requirements.

Comment 5: Limited description of salivary flow assessment.

Response:

The Methods section has been expanded to provide a detailed description of saliva collection. We now clarify that unstimulated whole saliva was collected under standardized conditions at all four measurement time points. We further explain that salivary flow rate was not measured because the primary objective was the determination of calcium concentration rather than total calcium secretion. This limitation has been explicitly acknowledged in the Discussion, where we recommend inclusion of salivary flow measurements in future studies.

Comment 6: Numerous unsupported statements in the Introduction and Discussion.

Response:

The Introduction and Discussion have been carefully revised to remove unsupported or overly speculative statements. Several sections were rewritten to distinguish clearly between established evidence from the literature and interpretations derived from the present findings. Additional references were incorporated where appropriate, while speculative mechanistic explanations are now explicitly identified as hypotheses requiring further investigation.

Comment 7: Table 2 is misleading and does not accurately represent the crossover study design.

Response:

We fully agree with this comment. The original figure and accompanying table have been redesigned to clearly illustrate the three-treatment, three-period crossover design. The revised study flow diagram now demonstrates that all 48 participants received each of the three toothpaste formulations according to one of the randomized treatment sequences (ABC, BCA, or CAB), separated by standardized 48-hour washout periods. The revised figure also illustrates participant progression through all treatment periods and saliva collection time points (T0–T3), thereby avoiding the previous impression of three independent parallel groups.

Response to Reviewer #1

We sincerely thank Reviewer #1 for the thorough and constructive evaluation of our manuscript. We appreciate the positive assessment of the scientific relevance of the study and the many valuable suggestions for improving the methodological transparency and statistical analysis. We have carefully considered every comment and have substantially revised the manuscript accordingly. Our detailed responses are provided below.

General comment

The manuscript presents analysis of data generated from a randomized cross-over clinical study evaluating the short-term effects of three commercially available toothpaste formulations on total salivary calcium concentrations in healthy young adults. The topic is relevant, the study is generally clearly written, the experimental procedures are described in detail...

Response:

We sincerely thank the reviewer for the positive evaluation of the study and for recognizing the relevance of our research question. We appreciate the constructive suggestions, which have substantially improved the clarity, methodological rigor, and statistical reporting of the manuscript.

Comment 1

Methods reporting needs improvement. The manuscript should follow the CONSORT guidelines and use separate subsections such as Trial design, Participants, Interventions, Outcomes, Sample size, Randomization, Blinding, Statistical analysis, etc.

Response:

We agree with this important recommendation. The Methods section has been completely reorganized following the CONSORT 2010 Statement and the CONSORT extension for randomized crossover trials where applicable. Separate subsections have been introduced for Ethics, Sample Size, study design, participant recruitment, Randomization, interventions, outcome measures; saliva collection, laboratory analysis, and statistical analysis. Redundant information has been removed, and the methodological description has been substantially expanded to improve transparency and reproducibility.

Comment 2a

Randomization and allocation concealment should be described separately.

Response:

Thank you for this suggestion. The manuscript now clearly distinguishes between sequence randomization and allocation concealment. The randomized treatment sequences were generated before participant recruitment by an independent investigator who was not involved in enrolment or outcome assessment. Allocation was concealed until participant assignment, and investigators performing the laboratory analyses remained blinded to treatment allocation until completion of data analysis.

“Randomization. Participants were assigned to the treatment sequences using a computer-generated randomization list with a 1:1 allocation ratio. The randomization sequence was generated prior to participant enrollment by an investigator who was not involved in participant recruitment or outcome assessment.”

“Allocation concealment. Allocation concealment was ensured by using sequentially numbered, opaque, sealed envelopes containing the treatment assignments. The envelopes were opened only after a participant had been enrolled, thereby preventing foreknowledge of the assigned treatment sequence.”

Comment 2b

More details on the randomization procedure are needed.

Response:

Additional details have been incorporated into the Methods section. The revised manuscript now describes the three-sequence Latin-square crossover design (ABC, BCA, and CAB), the generation of the randomization list, participant allocation, and the implementation of the randomized treatment sequences.

Comment 3a

Repeated-measures ANOVA does not adequately account for sequence, period, and carry-over effects.

Response:

We fully agree and thank the reviewer for this important observation. Following this recommendation, the statistical analysis has been completely revised. The previous repeated-measures ANOVA has been replaced by a linear mixed-effects model estimated by restricted maximum likelihood (REML). Toothpaste formulation, measurement time, treatment sequence, and the toothpaste-by-time interaction were included as fixed effects, whereas participant was included as a random intercept. Treatment sequence was retained in the final model to account for possible sequence-related effects. Because of the characteristics of the three-sequence Latin-square design, period and sequence were collinear and could not be entered simultaneously into the same model. This issue is now explicitly explained in the Methods section, and sensitivity analyses showed no evidence of clinically meaningful carry-over effects.

Comment 3b

A linear mixed-effects model would have been more appropriate.

Response:

We appreciate this recommendation and have implemented it in the revised manuscript. The entire statistical analysis has been repeated using linear mixed-effects modelling. The Results section has been rewritten accordingly and now includes fixed-effect parameter estimates, Type III tests of fixed effects, estimated marginal means with 95% confidence intervals, Bonferroni-adjusted pairwise comparisons, and covariance parameter estimates.

Comment 3c

Model assumptions were not assessed.

Response:

Thank you for highlighting this point. Model assumptions were evaluated by visual inspection of residual histograms, normal Q–Q plots, and residual-versus-fitted plots. No substantial deviations from normality or homoscedasticity were observed. This information has been added to the Results section.

Comment 3d

Independent-samples tests are inappropriate for a crossover design.

Response:

We agree. Independent-samples analyses have been removed from the manuscript. All statistical analyses are now based on the linear mixed-effects model, which appropriately accounts for repeated observations within participants and the crossover study design. Pairwise comparisons were performed using estimated marginal means derived from the fitted model with Bonferroni adjustment for multiple testing.

Comment 3e

Confidence intervals should be incorporated into the interpretation.

Response:

We appreciate this suggestion. Throughout the revised Results and Discussion, treatment effects are now interpreted using both effect estimates and their corresponding 95% confidence intervals rather than relying solely on P values. This revision provides a more balanced assessment of the magnitude and precision of the observed effects and is consistent with current recommendations for reporting clinical studies.

Comment 4a

Statements of significance should always be accompanied by P values.

Response:

The Results section has been carefully revised. All statements referring to statistically significant findings are now accompanied by the corresponding P values.

Comment 4b

The conclusions should acknowledge that the study population consisted only of participants from Witten/Herdecke University.

Response:

We agree and have revised both the Discussion and the Conclusion accordingly. We now explicitly state that the study population consisted exclusively of healthy volunteers recruited from Witten/Herdecke University and acknowledge that this may limit the generalizability of the findings. We further emphasize that larger multicentre studies including more diverse populations are required to confirm the present results.

Response to Reviewer #2

We sincerely thank Reviewer #2 for the thorough evaluation of our manuscript and for the many constructive comments and suggestions. We appreciate the detailed feedback, which has substantially improved the quality, clarity, and scientific rigor of our manuscript. Each comment has been carefully addressed below, and all corresponding changes have been incorporated into the revised manuscript.

Response to Reviewer #3

We sincerely thank Reviewer #3 for the careful review of our manuscript and for the constructive suggestions. We appreciate the positive evaluation of our work and have revised the manuscript accordingly. All comments have been addressed individually below, and the corresponding revisions have been incorporated into the manuscript.

Attachment Submitted filename: Response to the Academic Editor_Reviewer_15 7 2026.docx

# Peer review: Decision Letter 1

**DOI:** 10.1371/journal.pone.0358715.r003

**Authors:** Esra Cengiz Yanardag

1 Sep 2026

Short-term effects of different toothpaste formulations on total salivary calcium: a randomized cross-over study

PONE-D-26-16082R1

Dear Dr. Aminy,

We’re pleased to inform you that your manuscript has been judged scientifically suitable for publication and will be formally accepted for publication once it meets all outstanding technical requirements.

Within one week, you’ll receive an e-mail detailing the required amendments. When these have been addressed, you’ll receive a formal acceptance letter and your manuscript will be scheduled for publication.

An invoice will be generated when your article is formally accepted. Please note, if your institution has a publishing partnership with PLOS and your article meets the relevant criteria, all or part of your publication costs will be covered. Please make sure your user information is up-to-date by logging into Editorial Manager at Editorial Manager® and clicking the ‘Update My Information' link at the top of the page. For questions related to billing, please contact billing support.

If your institution or institutions have a press office, please notify them about your upcoming paper to help maximize its impact. If they’ll be preparing press materials, please inform our press team as soon as possible -- no later than 48 hours after receiving the formal acceptance. Your manuscript will remain under strict press embargo until 2 pm Eastern Time on the date of publication. For more information, please contact onepress@plos.org.

Kind regards,

Esra Cengiz Yanardag

Academic Editor

PLOS One

Additional Editor Comments (optional):

Reviewers' comments:

Reviewer's Responses to Questions

**Comments to the Author**

Reviewer #1: All comments have been addressed

Reviewer #3: All comments have been addressed

**********

2. Is the manuscript technically sound, and do the data support the conclusions?

Reviewer #1: (No Response)

Reviewer #3: Yes

**********

3. Has the statistical analysis been performed appropriately and rigorously?

Reviewer #1: (No Response)

Reviewer #3: Yes

**********

4. Have the authors made all data underlying the findings in their manuscript fully available?

The PLOS Data policy

Reviewer #1: (No Response)

Reviewer #3: Yes

**********

5. Is the manuscript presented in an intelligible fashion and written in standard English?

Reviewer #1: (No Response)

Reviewer #3: Yes

**********

Reviewer #1: (No Response)

Reviewer #3: (No Response)

**********

what does this mean?). If published, this will include your full peer review and any attached files.

If you choose “no”, your identity will remain anonymous but your review may still be made public.

**Do you want your identity to be public for this peer review?** For information about this choice, including consent withdrawal, please see our Privacy Policy

Reviewer #1: No

Reviewer #3: No

**********

# Editor report: Acceptance letter

**DOI:** 10.1371/journal.pone.0358715.r004

**Authors:** Esra Cengiz Yanardag

PONE-D-26-16082R1

PLOS One

Dear Dr. Aminy,

I'm pleased to inform you that your manuscript has been deemed suitable for publication in PLOS One. Congratulations! Your manuscript is now being handed over to our production team.

At this stage, our production department will prepare your paper for publication. This includes ensuring the following:

* All references, tables, and figures are properly cited

* All relevant supporting information is included in the manuscript submission,

* There are no issues that prevent the paper from being properly typeset

You will receive further instructions from the production team, including instructions on how to review your proof when it is ready. Please keep in mind that we are working through a large volume of accepted articles, so please give us a few days to review your paper and let you know the next and final steps.

Lastly, if your institution or institutions have a press office, please let them know about your upcoming paper now to help maximize its impact. If they'll be preparing press materials, please inform our press team within the next 48 hours. Your manuscript will remain under strict press embargo until 2 pm Eastern Time on the date of publication. For more information, please contact onepress@plos.org.

You will receive an invoice from PLOS for your publication fee after your manuscript has reached the completed accept phase. If you receive an email requesting payment before acceptance or for any other service, this may be a phishing scheme. Learn how to identify phishing emails and protect your accounts at https://explore.plos.org/phishing.

If we can help with anything else, please email us at customercare@plos.org.

Thank you for submitting your work to PLOS One and supporting open access.

Kind regards,

PLOS One Editorial Office Staff

on behalf of

Dr. Esra Cengiz Yanardag

Academic Editor

PLOS One
